# Low-Grade Ovarian Stromal Tumors with Genetic Alterations of the Wnt/β-Catenin Pathway That Is Crucial in Ovarian Follicle Development and Regulation

**DOI:** 10.3390/cancers14225622

**Published:** 2022-11-16

**Authors:** Gloria Zhang, Chad M. Michener, Bin Yang

**Affiliations:** 1 Pathology and Laboratory Medicine Institute, Cleveland Clinic Lerner College of Medicine, Cleveland, OH 44195, USA; 2 Obstetrics, Gynecology and Women’s Health Institute, Cleveland Clinic Lerner College of Medicine, Cleveland, OH 44195, USA

**Keywords:** Wnt pathway, beta-catenin, ovary, stromal tumor, solid peudo-papillary tumor, microcystic stromal tumor, signet ring stromal tumor

## Abstract

**Simple Summary:**

The Wnt signaling pathway plays an important role in the normal development and regulation of ovarian follicles. Genetic or epigenetic activation of β-catenin has been implicated in tumorigenesis of a spectrum of ovarian neoplasms. This review summarizes the recent findings of the β-catenin mutations in a family of low-grade ovarian stromal tumors.

**Abstract:**

The Wnt signaling pathway is important in the normal development and regulation of ovarian follicles throughout the lifecycle of females. Dysregulation of the Wnt signaling pathway, genetically or epigenetically, with subsequent activation of β-catenin has been implicated in tumorigenesis of a spectrum of ovarian neoplasms, from benign to malignant. We review the recent findings of the Wnt signaling pathway involved in regulating normal physiologic processes of the ovarian follicle cycle. We also review the β-catenin mutations in a family of low-grade ovarian stromal tumors, focusing on characterizing their shared morphological features and the utility of immunohistochemistry of β-catenin in facilitating the accurate diagnosis of these ovarian stromal tumors. The Wnt signaling pathway is one of the most critical mechanisms in regulating cell proliferation, differentiation, and morphogenesis. The Wnt signaling pathway comprises a diverse group of glycoproteins that serve as ligands and bind to transmembrane Frizzled family receptors. The ligand-receptor interactions activate the pathway and govern the downstream signaling cascades, ultimately affecting the transcriptional control of the cellular cytoskeleton, organelle dynamics, epithelial-mesenchymal interaction, and tissue remodeling in the ovary. Wnt signaling consists of two major pathways: a canonical pathway that is β-catenin-dependent and a non-canonical Wnt pathway that is β-catenin-independent. Canonical Wnt signaling is governed by the interaction of β-catenin with other molecules to regulate cellular decisions related to proliferation and differentiation. Recent studies have demonstrated that the Wnt signaling pathway plays important roles in the development and regulation of ovarian folliculogenesis and oogenesis.

## 1. Wnt Pathway in the Development and Regulation of Ovarian Follicle Cycle

The Wnt pathway is critical to the development and function of the ovary in three aspects: (1) it is essential during the embryonic development of the ovary; (2) it is crucial for the development and regulation of ovarian follicles from birth to maturity; and (3) it plays an essential role in steroid hormone production which in turn regulates the development of ovarian follicles and fertility in the adult ovary.

### 1.1. Wnt Pathway during the Embryonic Development of the Ovary 

During embryonic development, the Wnt pathway plays an essential role in early ovarian development and suppression of the male reproductive tract. In the mammalian embryo, both sexes are initially indistinguishable morphologically. Specific hormones secreted by the differentiating embryonic testes, such as Mullerian inhibiting substance and testosterone, are required for sex-specific development. These hormones suppress females (Mullerian) but promote male (Wolffian) reproductive duct development. The signaling molecule Wnt-4 is essential for ovarian differentiation and oogenesis by stimulating granulosa cell differentiation and promoting female sexual development. At birth, sexual development in males with Wnt-4 mutation appears normal; however, Wnt-4-mutant females are masculinized with the absence of the Mullerian duct while developing the Wolffian duct. Wnt-4 null females have sex-reversed ovaries but with a significantly reduced number of oocytes and the expression of the genes associated with testicular development at birth. It has been shown that Wnt-4 suppresses the growth of Leydig cells in the developing ovary [1]. 

### 1.2. Synchronized Effort between the Wnt Pathway and Hormones in the Adult Ovary

The adult ovary undergoes constant dynamic changes throughout the estrous cycle as follicles progress from immature preantral follicles to more developed preovulatory follicles and eventually corpus luteum formation following ovulation. The complex process of folliculogenesis relies on synchronized efforts of hormones from the hypothalamus, pituitary, and gonads. While the initial stage of the follicles develops primarily in the absence of gonadotropin input, the transition from preantral to a preovulatory follicle occurs as a result of increased follicle-stimulating hormone (FSH) and luteinizing hormone (LH) responsiveness as well as numerous other local hormones and growth factors [2]. It has been shown that these hormones and the Wnt signaling pathway are mutually dependent on each other. The function of the gonadotropins depends on the presence of Wnt signaling pathways in a cell-specific manner at defined stages of follicular growth [3,4]. Several Wnt family members are expressed at different stages of the follicle development within the adult ovaries of humans and other mammals [5]. Conversely, Wnt family genes are transcriptionally regulated in response to the hormone input within the ovary. For example, the expression of Wnt-4 was increased in mouse granulosa cells following HCG stimulation, and a high level of Wnt-4 expression was detected in terminally differentiated luteal cells [6]. These data indicate that the synchronized efforts between the Wnt signaling pathway and hormones are fundamentally required for normal ovarian function and fertility in the adult ovary [7].

### 1.3. Role of β-Catenin in Steroid Production and Regulation 

Recent studies show that β-catenin is required to produce and regulate estrogen levels stimulated by FSH. The accumulation of β-catenin protein has been identified in high estrogen-producing follicles compared to those with low intrafollicular estradiol concentrations [8]. Evidence shows that the FSH level directly affects the accumulation of β-catenin protein in granulosa cells [9]. Activation of β-catenin by FSH is via stimulating the phosphorylation of β-catenin on Ser552 and Ser675 [10]. Furthermore, FSH-induced β-catenin activation promotes TCF-dependent transcriptional activity and the expression of downstream effector genes such as *NR5A1* and *Lhcgr* [10]. Together these data demonstrate that activation of β-catenin plays a pivotal role in FSH-mediated actions in ovarian follicular cells. Similar mutual effects have been observed in between the function of Wnt signaling pathway and LH-mediated progesterone production from bovine corpora luteum. A recent study shows that LH can activate β-catenin through the phosphorylation and inhibition of GSK3β. Conversely, transcriptionally active β-catenin can enhance the synthesis and expression of progesterone [11].

## 2. Ovarian Stroma and Stromal Cell Types

The ovarian cortical and medulla stroma is similar in appearance and continuous with ill-defined boundaries. Ovarian stroma in reproductive-age women is highly cellular and vascular with inconspicuous supporting fibers such as reticular fiber and collagen. It comprises a mixed population of incompletely characterized stromal cells or interstitial cells typically arranged in a dense storiform pattern [12]. Stromal cells associated with maturing follicles may secrete estrogen and acquire endocrine function. Details regarding the development and function of these stromal cells are largely unknown. Recently using single-cell RNA sequencing, investigators have identified approximately seven cell types and multiple cell clusters in the ovarian stroma [13,14]. The different subtypes of stromal cells have slightly different distributions within the ovary (e.g., cortex vs. medulla and hilus vs. non-hilus). The stromal cell distribution also appears to be cyclic and dynamic, likely affected by the follicle cycle. Changes are also evident over the reproductive lifespan, such as increased fibrotic collagen in aging ovaries [13,15]. Other molecular studies have identified cellular biomarkers highly expressed in different clusters of ovarian stromal cells, such as TCF21, COL1A2, GNL3, ARID5b, COUP-TFII, ARX, DCN and LUM [13,14,16]. Other cellular biomarkers that are recently identified in ovarian stromal cells include PDGFRA, DCN, COL1A1, COL6A1, STAR, and CYP17A1 [17,18]. However, the biological functions of these biomarkers are largely unknown. Using spatial transcriptome sequencing at different time points of follicular development, it is found that the spatial location of stromal cells is closely related to the stages of follicular development [19]. Based on these data, it is speculated that ovarian stromal cells are likely to transmit signals between different groups of cells, sending signals to granulosa cells and oocytes during follicle development.

During follicle maturation, the granulosa cells proliferate to form many layers around the oocyte. A boundary layer, the zona pellucida, appears between the oocyte and the granulosa cells. Stromal cells surrounding the follicle differentiate into the theca interna and the theca externa. Cells of the theca interna become cuboidal steroid-producing cells which often contain cytoplasmic lipid droplets. Androgens from thecal cells is converted to estrogen in granulosa cells and then released into circulation. The theca externa, a mixture of fibroblasts and smooth muscle cells lacking endocrine function, is contiguous with the surrounding ovarian stroma [20,21]. Recent studies show that murine theca cells arise from two types of progenitors: Gli1-positive and WT1-positive stromal cells [22]. The steroidogenic androgen-producing theca cells may arise from the mesonephros-derived Gli1-positive progenitors. In contrast, the theca fibroblasts, perivascular smooth muscle cells, and interstitial ovarian cells may arise from the ovarian WT1+ progenitors [22]. It is well known that stromal-epithelial cell interactions are necessary for tumor development, growth, angiogenesis, and metastasis. In a recent publication by Fujisawa et al., FOXL2, a vital factor for the maintenance of granulosa cell function and follicle cycle, was found to be expressed in some stromal cells of both primary and secondary ovarian epithelial cancers [23]. 

## 3. Activation of Wnt/β-Catenin Signaling Pathway in Gynecological Neoplasms

The β-catenin protein is stabilized and anchored to the cell membrane by the APC/Axin/GSK-3β complex, the so-called β-catenin destruction complex (1). When the Wnt pathway is activated during tumorigenesis, β-catenin is released and redistributed from the cell membrane to the nuclei and cytoplasm of tumor cells [24]. Excessive β-catenin activity can be achieved either by genetic mutations or epigenetic alterations of the critical components in the Wnt signaling pathway. In most human cancers, inactivation of the β-catenin destruction complex via mutation or promoter methylation of the *APC* and *Axin* genes is far more common than activating mutations of the β-catenin gene *CTNNB1*. Only approximately 3% of malignant neoplasms harbor *CTNNB1* mutations [25]. Moreover, somatic *APC* mutations are found in more than 80% of colorectal cancers, but only approximately 5% of colorectal cancers harbor *CTNNB1* mutation [24,25]. The Wnt pathway has been activated in many ovarian epithelial carcinomas [26,27]. However, only a small percentage of ovarian tumors harbor *CTNNB1* mutations [26,28]. Genetic alteration of β-catenin in ovarian epithelial cancers is linked to specific cell types [24]. *CTNNB1* mutations have been identified in 16–54% of endometrioid adenocarcinoma, which accounts for less than 10% of all ovarian carcinomas. However, *CTNNB1* mutations are rarely seen in other histologic types, such as serous, clear cell, and mucinous carcinomas [29,30].

The β-catenin protein is composed of a 23.2 kb polypeptide encoded by the *CTNNB1* gene, which has 16 exons and maps to 3p21 [31]. The primary structure of β-catenin protein consists of three domains: N-terminal domain, C-terminal domain and central armadillo repeat domain [32]. Generally, the N-terminal domain is the phosphorylation site for GSK-3β, an important protein leading to the degradation of the β-catenin protein. The C-terminal domain is associated with some nuclear effectors, such as TCF/LEF. The CTNNB1 mutations detected in human neoplasms are frequently missense mutations, and nearly all of them have been localized in exon 3, the phosphorylation sites for GSK-3β [24,31]. Thus far, most human neoplasms with frequent *CTNNB1* mutations are relatively low-grade with relatively favorable prognosis, such as desmoid tumor, renal angiomyolipoma, pulmonary lymphangioleiomyomatosis, and pancreatic solid pseudopapillary tumor [31,32].

## 4. The Family of Low-Grade Ovarian Stromal Tumors with β-Catenin Alterations

Recently mutations in the *CNNB1* gene have been detected in a family of low-grade stromal tumors of the ovary, which have overlapping morphological features, similar clinical presentation, and favorable prognosis. This family includes primary ovarian solid pseudopapillary neoplasm (SPPN), microcystic stromal tumor (MCST), and signet-ring stromal cell tumor (SRSCT). Table 1 compares the clinical and pathological features, immunohistochemical characterization, and molecular alterations of *CTNNB1* and *APC* genes in ovarian SPPN, MCST and SRSCT.

### 4.1. Solid Pseudopapillary Neoplasm (SPPN)

**Figure 1:**
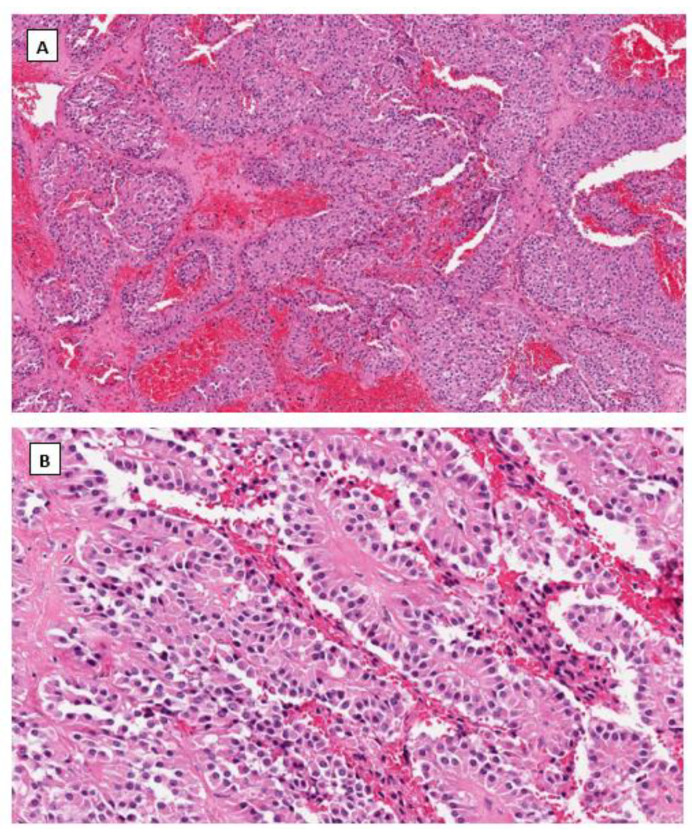

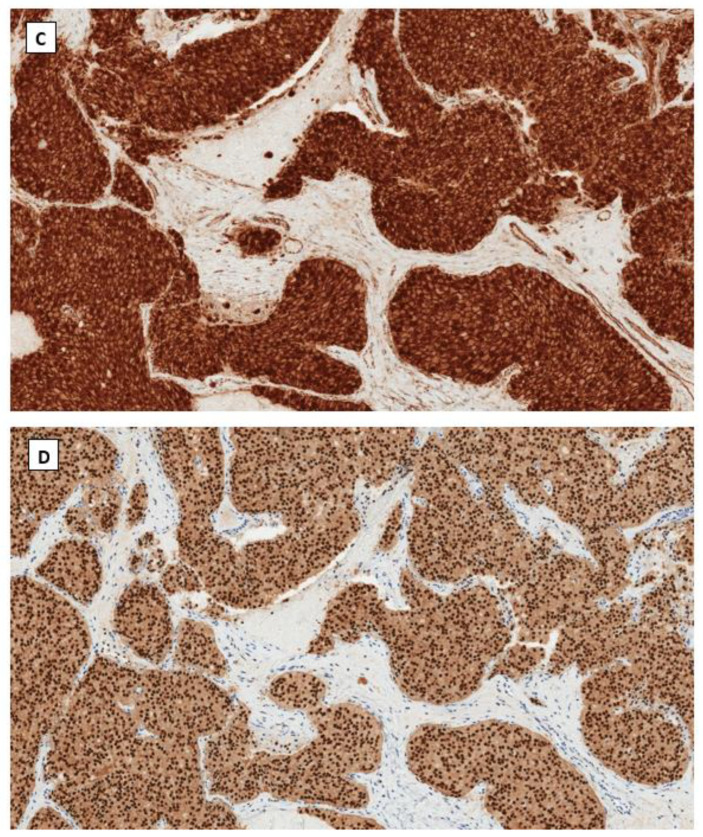

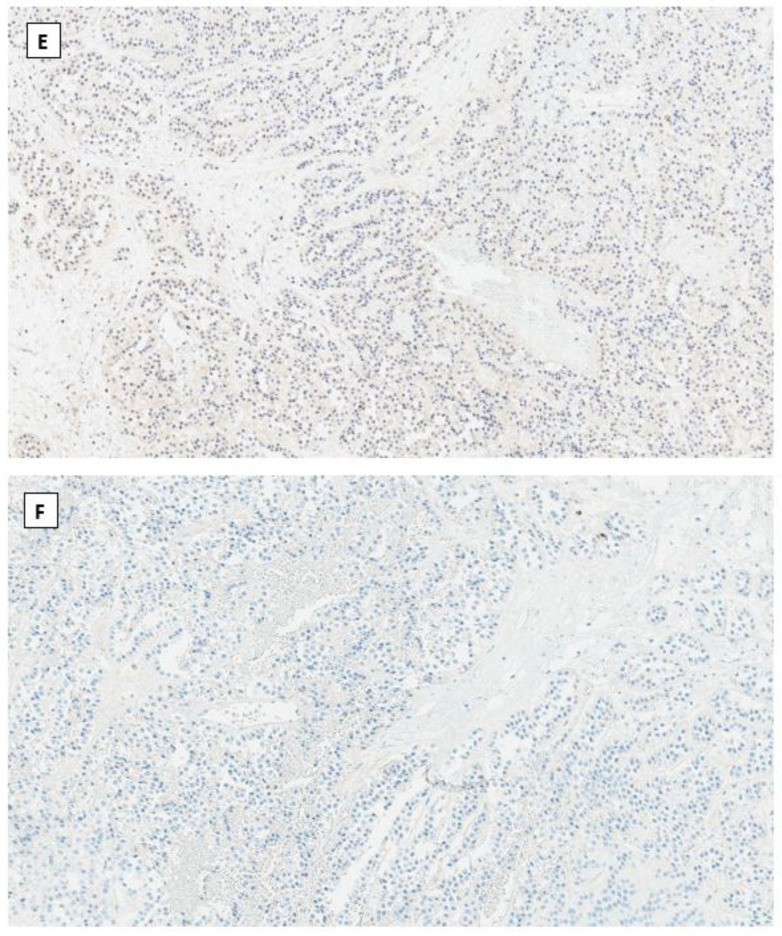
Solid pseudopapillary neoplasm of the ovary. Tumor cells grow in cords and pseudopapillary patterns with blend cytology and abundant delicate vasculature (H&E staining at 40× magnification for (**A**) and 200× magnification for (**B**)). Immunohistochemical staining demonstrates intense nuclear and cytoplasmic staining of β-catenin (**C**), intense nuclear with weak cytoplasmic staining of LEF-1 (**D**), and negative staining for SF1 (**E**) and inhibin (**F**). Immunostaining for β-catenin is at 200×, and for LEF-1, SF1 and inhibin are at 100× magnifications, respectively.

Dr. Young’s group first described SPPN in 2010 [33]. SPPN is grossly a solid and cystic tumor. When the cystic structure with hemorrhage is predominant, it can clinically mimic a benign hemorrhagic luteal cyst [34]. Microscopically the tumor shows solid, nested, and pseudopapillary growth patterns. Tumor cells are monotonous with round to oval nuclei, pale chromatin, and abundant cytoplasm that contains clear intracytoplasmic vacuoles or eosinophilic globules in some neoplastic cells. Cytological atypia is minimal (Figure 1). Differential diagnoses include sex cord-stromal tumor, steroid cell tumor and low-grade neuroendocrine tumor. Immunohistochemical studies reveal that tumor cells are positive for CD10, CD56 and vimentin but negative for cytokeratin (AE1/3 and CAM5.2), sex cord-stromal markers (SF1, inhibin, and calretinin), neuroendocrine biomarkers (synaptophysin, chromogranin and INSM1) and hormone receptors (ER, PR and AR). The most striking immunohistochemical feature is the diffuse nuclear and cytoplasmic immunoreactivity with β-catenin (Figure 1). In 2014, Kominami et al. first detected *CTNNB1* mutation at exon 3 in ovarian SPPN [35]. Since then, genetic alteration of β-catenin has been consistently identified in reported cases of SPPN. Currently, immunohistochemical staining for β-catenin with nuclear and cytoplasmic staining patterns has become the required criteria for accurate diagnosis of SPPN. 

### 4.2. Microcystic Stromal Tumor (MCST) 

**Figure 2:**
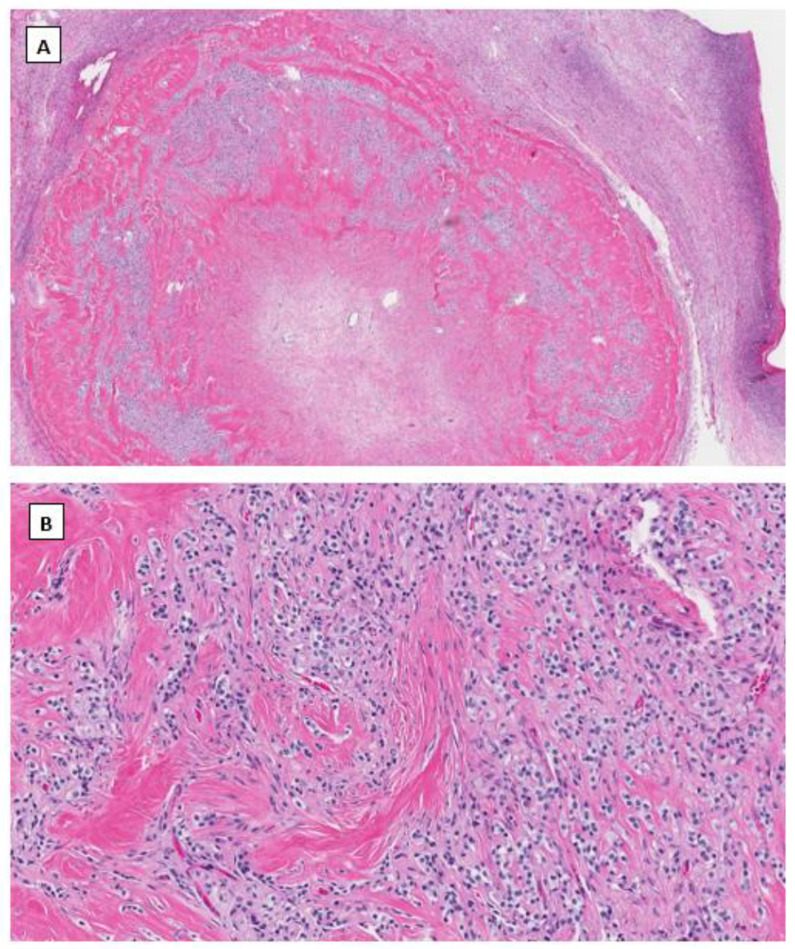

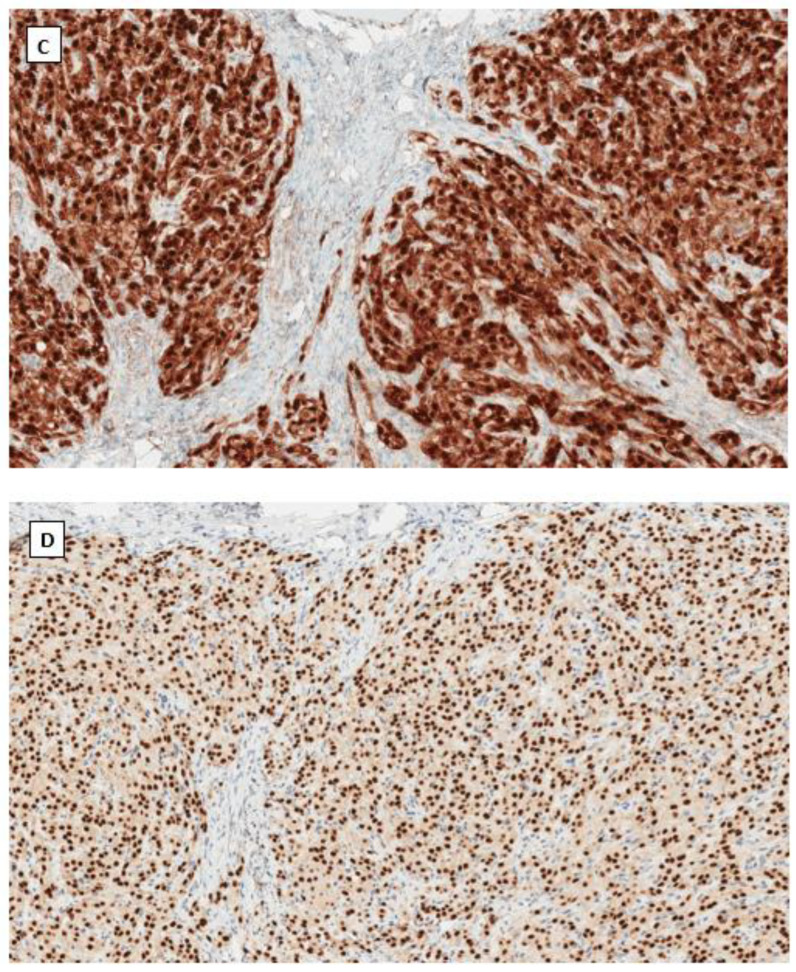

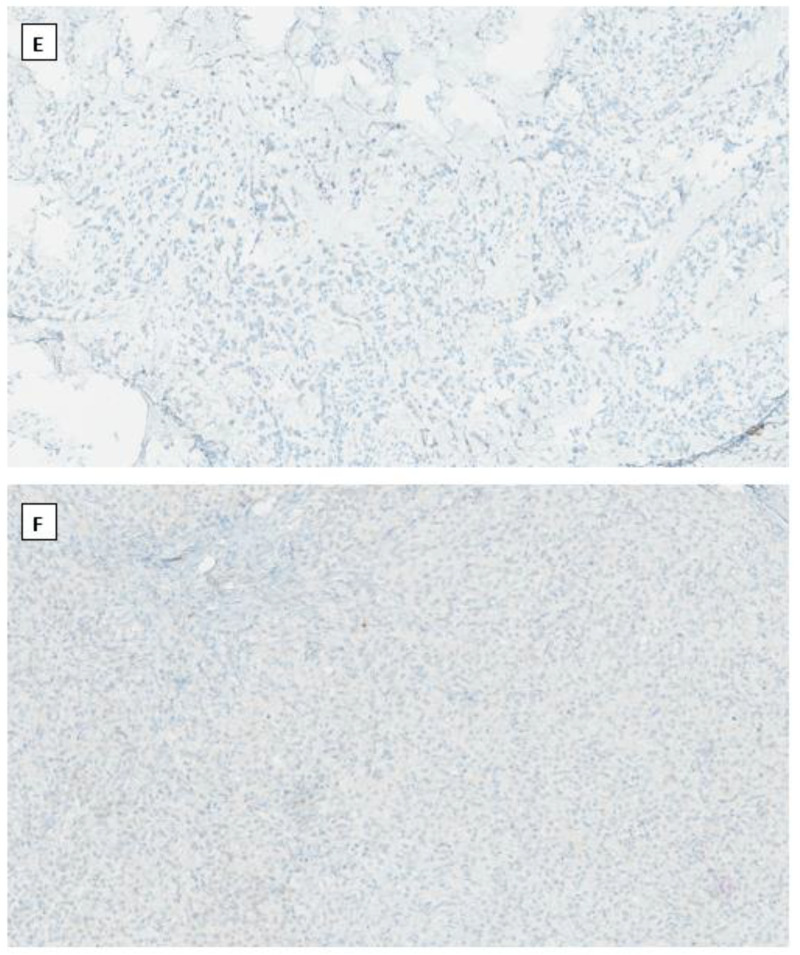
Microcystic stromal tumor of the ovary. Tumor cells grow in delicate cords or solid sheets with blend cytology in a background of extensive hyalinized stromal fibrosis (H&E stain at 20× magnification for (**A**) and 200× magnification for (**B**)). Immunohistochemical staining demonstrates intense nuclear and cytoplasmic staining of Beta-catenin (**C**), intense nuclear with weak cytoplasmic staining of LEF-1 (**D**), and negative staining for SF1 (**E**) and inhibin (**F**). Immunostaining for β-catenin is at 200×, and for LEF-1, SF1 and inhibin are at 100× magnifications, respectively.

In 2009, Irving J.A. and Young R.H. described a novel and rare ovarian stromal neoplasm and named it the microcystic stromal tumor [36]. MCST presents as a unilateral solid tumor in middle-aged women without hormonal manifestations. Microscopically, most MCSTs show a distinctive triad of histologic features, including microcystic change, solid cellular regions, and extensive hyalinized fibrous stroma. Tumor cells have round to oval nuclei with inconspicuous nucleoli and the eosinophilic cytoplasm (Figure 2). Besides the unique extensive hyalinized fibrous stroma, the cytological features of these tumor cells are similar to those seen in ovarian SPPN. Immunohistochemical staining shows that tumor cells are positive for CD10, cyclin D1 and vimentin and variably positive for WT1 and CD56. Tumor cells are usually negative for cytokeratin, epithelial membrane antigen, synaptophysin, chromogranin, INSM1, inhibin, calretinin, and SF1. Maeda et al. first identified an aberrant β-catenin nuclear and cytoplasmic accumulation in MCST cells immunohistochemically [37]. Further molecular studies showed the identification of *CTNNB1* mutations in exon 3, identical to that of SPPN, indicating dysregulation of the Wnt/β-catenin pathway in the pathogenesis of this neoplasm. Several other studies subsequently confirmed the above findings [38,39,40,41]. Furthermore, recent studies indicate that the incidence of MCST has increased among patients affected by familial adenomatous polyposis (FAP) due to *APC* germline mutations [42,43]. In both MCST cases reported by Liu et al. [43] and Lee et al. [42] there is no *CTNNB1* mutation identified besides *APC* mutations. This has prompted a comprehensive molecular study by McCluggage’s group with the largest case series of MCST thus far [44]. Among 10 cases of MSCTs, they identified 8 cases harboring *CTNNB1* mutations and 2 cases having *APC* mutations including one with germline *APC* mutation. *CTNNB1* and *APC* mutations are mutually exclusive with no neoplasm exhibiting a mutation in both [44]. 

### 4.3. Signet Ring Stromal Cell Tumor (SRSCT)

SRSCT is a rare ovarian stromal tumor that Ramzy first described in 1976 [45]. Thus far, only a dozen cases have been reported in the English literature [46,47,48,49,50]. Most SRSCT presented as a unilateral solitary ovarian tumor in middle-aged women with a mean age of 53. Like SPPN and MCST, almost all cases have a favorable prognosis. Grossly the tumor is sharply circumscribed with a thick capsule. Microscopically, SRSCT is characterized by the proliferation of stromal spindle cells merged with round cells containing eccentric nuclei and single vacuoles, resulting in a signet ring cell-like appearance. Signet ring stromal cells comprise approximately 20–70% of the neoplasm. The intracytoplasmic vacuoles are considered degenerative since they do not contain any mucin, lipid or glycogen. Some intracytoplasmic vacuoles can be calcified, giving a psammoma body-like appearance [51]. Tumor cells have no to minimal nuclear atypia with low mitotic activity. Immunohistochemically, tumor cells are positive for CD10, cyclin D1 and vimentin and variably positive for WT1, FOXL2, and SF1. Tumor cells are usually negative for epithelial membrane antigen, pan-cytokeratin, smooth muscle actin, inhibin, and calretinin [51,52,53]. The pathogenesis of SRSCT of the ovary is largely unknown. However, some authors speculated that SRSCT might be derived from ovarian fibroma [54]. However, a recent study failed to identify reticulin fibers individually wrapping around tumor cells, the histochemical staining pattern typically seen in ovarian fibroma [51]. In another recently reported case, the aberrant nuclear β-catenin expression resulted from the oncogenic mutation of the *CTNNB1* gene at exon 3, along with a germline missense mutation in Janus kinase 3 (*JAK3*) gene [55,56]. JAK3 is a non-receptor tyrosine kinase and plays a crucial role in epithelial-mesenchymal transition through its interaction with β-catenin [56]. 

Similar to MCST, mutations of *APC* gene, but not the *CTNNB1* gene, has also been reported for SRSCT. Yoshikawa et al. recently described a variant of SRSCT with the nuclear accumulation of β-catenin in an adult Asian woman harboring germline APC mutation, but lack of *CTNNB1* mutations. Another unusual case of SRSCT was reported recently by Chen et al. [57]. In contrast to the above reports, neither the nuclear accumulation of β-catenin protein nor mutation at the *CTNNB1* gene was identified in Chen’s case using next-generation sequencing with a panel of 54 genes. Arguably the tumor in the latter was less typical for SRSCT, given its bilateral presentation and different immunophenotype compared to the previous reports. Immunohistochemical study in Chen’s case showed that tumor cells are diffusely positive for SF1, calretinin and SMA, and focally positive for cytokeratin AE1/3 and ER. Tumor cells are also negative for cyclin D1 [57]. The reports on SRSCT appear less consistent in clinical presentation, pathological characterization and immunophenotype compared to MCST and SPPN. More cases with further molecular tests are needed to establish the consistent clinical presentation, pathological features and molecular makeup. 

### 4.4. Immunohistochemical Phenotype Shared in Ovarian Low-Grade Stromal Tumors

As mentioned above, the aberrant nuclear and cytoplasmic immunostaining pattern of β-catenin is the most striking immunohistochemical phenotype found in ovarian SPPN, MCST, and SRSCT. Immunohistochemical detection of nuclear and cytoplasmic accumulation of β-catenin has much higher sensitivity compared to molecular testing because mutations in several Wnt signaling genes, including *CTNNB1* and *APC*, can result in the same aberrant expression pattern of β-catenin protein [24,44,51]. 

Additionally, these three tumors also share similar immunohistochemical profiles of other markers/antibodies (Table 1). Immunoreactivities to CD10, CD56, cyclin D1, and vimentin have been consistently reported in most cases of these ovarian stromal tumors. Among them, cyclin D1 is one of the critical downstream effectors activated by β-catenin. Variable expression of WT1 suggests that these tumors may arise from WT1-positive stromal cells rather than Gli1-positive stromal cells. Lack of immunostaining for calretinin, inhibin, and SF1 in most cases indicates that these tumors are likely composed of pure stromal cells, different from other sex cord–stromal tumors such as granulosa cell tumors and Sertoli–Leydig cell tumors. Negative epithelial makers, including AE1/3, CAM5.2 and EMA, essentially exclude their epithelial origin. Morphologically, these tumor cells could be mistaken for carcinoid or other low-grade neuroendocrine tumors. It is further complicated by frequent immunoreactivity to CD56 in these ovarian stromal tumor cells. Therefore, a panel of antibodies, including synaptophysin, chromogranin and/or INSM1, shall be applied to exclude neuroendocrine tumors. 

LEF1 belongs to the T cell Factor (TCF)/LEF family of transcription factors and contains a highly conserved DNA-binding domain associated with the C terminal of β-catenin. LEF1 is one of the critical nuclear effectors downstream of the Wnt/β-catenin signaling pathway. When the Wnt signaling is inactivated, LEF1 is bound to Groucho-related co-repressors, negatively regulating the expression of Wnt signaling genes. Upon activation of the Wnt pathway via β-catenin mutation, mutant β-catenin protein displaces the Groucho-related co-repressors and promotes LEF1 transcription factor activity [58]. Compared to normal pancreatic tissue, nuclear overexpression of LEF1 has been identified in pancreatic SPPNs with high sensitivity and specificity [59]. However, the LEF1 expression level has not been well studied in ovarian low-grade stromal tumors harboring β-catenin mutations. We have studied the LEF1 expression, immunohistochemically with appropriate controls, in a limited number of ovarian low-grade stromal tumors (unpublished data). We found that nuclear LEF1 was overexpressed in both ovarian SFPN and MCST (Figure 1 and Figure 2). This provides additional supportive evidence of the activation of the Wnt pathway in these low-grade ovarian stromal tumors. 

In summary, the Wnt/β-catenin pathway plays an important role in ovarian follicular and stromal physiology. However, this pathway can become dysregulated, leading to a variety of ovarian neoplasms. Besides ovarian endometrioid adenocarcinoma, mutations in *CTNNB1* or *APC* genes have been identified in a family of low-grade ovarian stromal tumors, resulting in nuclear and cytoplasmic accumulation of β-catenin. Identification of the hotspot mutations in exon 3 of the *CTNNB1* gene indicates that the activation of the Wnt signaling pathway plays a crucial role in the tumorigenesis of these low-grade ovarian stromal tumors. The overlapping morphological features and shared immunophenotype seen in these low-grade ovarian stromal tumors suggest a likely common origin from the same type of ovarian stromal cells. 

## Figures and Tables

**Table 1:** Clinical, Pathological and Immunohistochemical Features and Genetic Alteration of β-catenin in Ovarian SPPN, MCST and SRST.

	SPPN	MCST	SRSCT
Clinicopathologic Features	Mean age 45	Mean age 43	Mean age 53
	Unilateral, solid and cystic	Unilateral, solid and well demarcated	Unilateral, solid and well demarcated
Morphological features			
Growth patterns	Solid, pseudopapillary and microcystic growth patterns	Solid and microcystic growth patterns	Solid, nested and trabecular growth patterns
Tumor cell Morphology	Uniform round nuclei, abundant eosinophilic cytoplasm with or without cytoplasmic vacuoles Lack of cytological atypia and mitosis	Uniform round nuclei, abundant eosinophilic cytoplasm with or without cytoplasmic vacuoles Lack of cytological atypia and mitosis	Signet ring-like cells with eccentric nuclei, and a single large cytoplasmic vacuole, admixed with spindle cells Lack of cytological atypia and mitosis
Immunophenotype			
Diffuse positive	CD10, vimentin, CD56, Cyclin D1	CD10, vimentin, CD56, Cyclin D1	CD10, vimentin, CD56, Cyclin D1
Variable positive	WT1, CD99, PR, CD117	WT1, CD99, SF1, FOXL2	WT1, CD99, SF1, FOXL2
Totally negative	AE1/3, CAM5.2, EMA, inhibin, calretinin, SMA, synaptophysin, INSM1	AE1/3, CAM5.2, EMA, inhibin, calretinin, SMA, synaptophysin, INSM1	AE1/3, CAM5.2, EMA, inhibin, calretinin, SMA, synaptophysin, INSM1
Mutations of Wnt signaling genes	*CTNNB1* exon 3	*CTNNB1* exon 3 or APC gene	*CTNNB1* exon 3 or APC gene
Immunohistochemical staining of β-catenin	Nuclear and cytoplasmic	Nuclear and cytoplasmic	Nuclear and cytoplasmic

Note: SPPN, solid pseudopapillary neoplasm; MCST, microcystic stromal tumor; SRST, Signet ring cell stromal tumor.
